# Identity, storytelling and the philanthropic journey

**DOI:** 10.1177/0018726714564199

**Published:** 2015-10

**Authors:** Mairi Maclean, Charles Harvey, Jillian Gordon, Eleanor Shaw

**Affiliations:** Newcastle University Business School, UK, mairi.maclean@newcastle.ac.uk; Newcastle University Business School, UK, Charles.Harvey@ncl.ac.uk; Glasgow University, UK, Jillian.Gordon@glasgow.ac.uk; Strathclyde Business School, UK, eleanor.shaw@strath.ac.uk

**Keywords:** generativity, identity narratives, identity work, narrative identity, philanthropic journey, storytelling

## Abstract

This article develops theoretical understanding of the involvement of wealthy entrepreneurs in socially transformative projects by offering a foundational theory of philanthropic identity narratives. We show that these narratives are structured according to the metaphorical framework of *the journey*, through which actors envision and make sense of personal transformation. The journey provides a valuable metaphor for conceptualizing narrative identities in entrepreneurial careers as individuals navigate different social landscapes, illuminating identities as unfolding through a process of wayfinding in response to events, transitions and turning-points. We delineate the journey from entrepreneurship to philanthropy, and propose a typology of rewards that entrepreneurs claim to derive from giving. We add to the expanding literature on narrative identities by suggesting that philanthropic identity narratives empower wealthy entrepreneurs to generate a legacy of the self that is both self- and socially oriented, these ‘generativity scripts’ propelling their capacity for action while ensuring the continuation of their journeys.

## Introduction

This article examines the journeys of entrepreneurial philanthropists as recounted in life-history interviews with UK-based entrepreneurs. It examines their giving within the context of their life stories (Atkinson, 1998; Denzin, 1989; Linde, 1993; McAdams, 1988, 1993), and evaluates the benefits they claim to derive from their philanthropy. Philanthropists’ self-narratives help to publicize the rewards of charitable giving – ‘the donation of money … that benefits others beyond one’s own family’ (Bekkers and Wiepking, 2011: 925) – to attract new donors to the cause (Kornberger and Brown, 2007; Martens et al., 2007). Such narratives shift the focus from the universal – too vast to address in a useful manner – to something more defined to which new donors can relate and potentially make a difference. In this sense, these stories emphasize agency, turning the spotlight on human powers to formulate agential projects (Archer, 2000; Emirbayer and Mische, 1998).

In making sense of the philanthropic journey, this article extends theoretical approaches to the study of contemporary entrepreneurial philanthropy – still at a pre-paradigmatic, embryonic stage – by laying the foundations for a theory of philanthropic identity narratives (Nicholls, 2010; Taylor et al., 2014). These are embedded in wider canonical discourses that exhort entrepreneurs to assume particular moralities, and shed light on the construction of desirable past and future selves (Pratt et al., 2006; Watson, 2008; Ybema, 2010). The acquisition of a philanthropic identity is experienced and narrated as a journey, which is primarily a quest for meaning (Gregg, 2006; Hytti, 2005; Karp, 2006). The journey metaphor elucidates processes of identity-building accomplished in the course of entrepreneurial careers. Changes in professional identities accompany changes in organizational life that are often unpredictable, meandering and discontinuous. This casts light on identities as evolving over time through a process of wayfinding in response to role changes, setbacks and turning-points (Ingold, 2000), as actors ‘make sense of and “enact” their environments’ (Pratt et al., 2006: 235; Weick, 1995). We draw on the notion of generativity (Erikson, 1950; Giacolone et al., 2008; McAdams, 1988, 1993), defined as ‘purposeful action for the well-being of future generations, and the emergence of individual purpose and agency’ (Creed et al., 2014: 113), to make a conceptual contribution to the burgeoning literature on narrative identities. We demonstrate that through giving and narrating their giving, philanthropists generate a legacy of the self, producing ‘generativity scripts’ that propel their capacity for agency in targeted communities while keeping their individual journeys going (Creed et al., 2014). Generativity scripts constitute ‘outlines for chapters yet to be lived’ that ‘perform something worthy to be remembered’ (McAdams, 1988: 252). As such, they concern both identity narratives and philanthropic projects, and play a key part in ‘individuals’ self-construction of generative selves’ (Creed et al., 2014: 113).

The present article is situated in the context of a wider investigation into the nature of contemporary entrepreneurial philanthropy, located within what Acs and Phillips (2002: 201) term the ‘entrepreneurship-philanthropy nexus’ (Anheier and Leat, 2006; Bishop and Green, 2008; Dees, 2008; Maclean et al., 2013; Shaw et al., 2013; Villadsen, 2007). Following Harvey et al. (2011: 428), we define entrepreneurial philanthropy as *the pursuit by entrepreneurs on a not-for-profit basis of social objectives through active investment of their economic, cultural, social and symbolic resources*. Entrepreneurial philanthropists are characterized by their drive to accumulate personal fortunes, together with a concomitant impulse to employ a share of their wealth in pursuit of philanthropic ventures over which they exercise control (Bandura, 1997). Hence, their focus is directed towards the (entrepreneurial) creation of wealth and the (philanthropic) redistribution of that wealth to serve specified social objectives (Acs and Phillips, 2002).

The 21st century has seen a progressive increase in inequality (Picketty, 2014). The incapacity of the state to meet growing welfare needs has facilitated the emergence of a neo-liberal ‘common sense’, which deems the private sphere more effective than the public in serving the ‘common interest’ (Swalwell and Apple, 2011: 369). Yet, despite rising interest in charitable giving, entrepreneurial philanthropy remains under-researched and under-theorized (Taylor et al., 2014). Given the growing discursive space accorded to philanthropy and volunteering in the welfare policies of western governments, this is in need of remedy (Ball, 2008; Villadsen, 2007). In particular, little is known about how philanthropists construct their philanthropic journeys, or about the use of narratives in shoring up this process (Downing, 2005; Politis, 2005). It is this research gap that the present article aims to address. We pose two key research questions. First, what do philanthropists say characterizes the personal change process or ‘journey’ they undergo in becoming philanthropic? Second, despite common perceptions of there being ‘no implied reciprocity or tangible reward for the donor’ (Radley and Kennedy, 1995: 688), what are the principal rewards, the *quid pro quo*, which they claim to derive from their involvement in philanthropy, and how might these be interpreted?

The article is organized as follows. The next section examines the relationship between narrative identities, storytelling and philanthropy. The following section is methodological, explaining our research process, data sources and analytical methods. In our two empirical sections, we draw on the rich data obtained in life-history interviews with entrepreneurial philanthropists to delineate the philanthropic journey, highlighting generic features that map to corresponding stages in the evolving philanthropic narrative identity. We then propose and evaluate a typology of the principal benefits philanthropists claim to derive from their giving. Finally, we discuss our findings, consider the implications for the theory and practice of entrepreneurial philanthropy, and reflect on the limitations of the study and possibilities for further research.

## Storytelling, narrative identity and philanthropy

Scholars of management and organization studies increasingly recognize that narratives and storytelling are critical to ways of organizing (Boje, 2008; Brown, 2006, 2014; Brown and Thompson, 2013; Brown et al., 2008; Czarniawska, 1998; Gabriel, 1995, 2000; Maclean et al., 2012; Rhodes and Brown, 2005). De Certeau (1984: 81) sees clear homologies between ways of storytelling and ways of acting: ‘The story does not express a practice … It *makes* it’. Ricoeur (1981) and Bruner (1990) suggest that storytelling can alter meanings and therefore actions, meaning-making being pivotal to human action. Considered thus, there is an explicit link between narrating and doing, sensemaking and action-making (Downing, 2005), and between entrepreneurs as doers and stories that *enact* social realities that had not materialized prior to their telling (Brown, 2006; Czarniawska, 1998). Stories evolve over time, highlighting ‘the process of storytelling as the never-ending construction of meaning’ (Czarniawska, 1998: 15). In this way, narration is implicated in the activation of agency, as agents ponder alternatives to their social contexts (Archer, 2000).

Stories are also *performative* (Brown, 2006; Goffman, 1959: 40), bringing saying and doing together in a dramatic realization by agential protagonists in the presence of others, through which storytellers fashion themselves as ‘characters’ (Downing, 2005; Martens et al., 2007: 1110). Hence, storytelling is bound up with *identity*, the way individuals elect to exhibit themselves (Brown and Jones, 2000; Goffman, 1959), and *identity work*, through which they carve out in discourse their sense of individuality (Brown, 2014; Snow and Anderson, 1987; Sveningsson and Alvesson, 2003; Watson, 2008) and ‘attach themselves to certain issues … to articulate and give meanings for themselves and their actions’ (Hytti, 2005: 599). Identity work forms a continuous, social process (Gergen, 1991). According to Ezzy (1998: 239), ‘a narrative identity provides a subjective sense of self-continuity as it symbolically integrates the events of lived experience in the plot of the story a person tells about his or her life’, often during dialogic encounters with others. Identity narratives are shaped and defined by the individual’s personalized perception of reality (Karp, 2006; Ricoeur, 1988). The self is hence fundamentally a ‘figured self – a self which figures itself as this or that’ (Ricoeur, 1991: 80). Authoring reflexively accomplished self-narratives allows actors to present to others favoured versions of the self, redefining their sense of who they are (Brown and Jones, 2000: Goffman, 1959; Kornberger and Brown, 2007; Sveningsson and Alvesson, 2003). Identity work is essentially twofold, some aspects implicating the personal sphere and others being oriented towards public consumption (Watson, 2008). As such, identity construction is bound up with ontological issues concerning the individual’s mode of being in society, involving both the public projection of identity and its private lived experience (Ybema et al., 2009).

The fashioning of narrative identities is a dynamic process constantly open to refiguration, implying transition and variability (Ezzy, 1998; Ricoeur, 1991). The authoring of self-narratives helps individuals to ‘reweave their webs of beliefs and their habits of action’ (Tsoukas and Chia, 2002: 577), inducing improvisations and allowing for the possibility of change in personal trajectories as they adapt to new experiences. In this sense, narrative identities are ‘infinitely revisable, and always provisional, works-in-progress’ (Brown, 2006: 740–741), never permanently stabilized but in ‘perpetual states of storied becoming’ (Brown and Thompson, 2013: 1153). Hence, it is possible to spin varied and even inconsistent narratives about our lives (Ricoeur, 1988). This said, the overriding objective in fashioning self-narratives is to replace fragments of tales that are incoherent or unacceptable with a coherent story indicative of self-constancy (Linde, 1993), so that narrators can ‘recognize themselves in the stories they tell about themselves’ (Ricoeur, 1988: 247).

Identities, like narratives, are ‘power effects’ (Kornberger and Brown, 2007: 500; Martens et al., 2007). Philanthropic engagement can therefore be conceived both as a form of identity work and as a *route to power and influence* in wider society (Ball, 2008; Breeze, 2007). For engaged philanthropists, giving goes hand-in-hand with social investment and influence to form a potent giving–investment–influence triad, through which they weave a web of connections (Bosworth, 2011; Swalwell and Apple, 2011). The road to influence is facilitated by the acquisition of legitimacy, which in turn depends on narrative construction (Lounsbury and Glynn, 2001; Middleton-Stone and Brush, 1996; Suchman, 1995). To have influence, narrators must be able to present themselves as having lived ‘a reasonable life’ (Habermas and Bluck, 2000: 751). This entails constructing a positive identity whereby the self is portrayed as virtuous and held in high regard (Dutton et al., 2010).

McAdams (1988: 260, 269) suggests that the creation of a positive identity that the individual can esteem forms an ‘action script for the future’ through which the individual re-evaluates ‘his or her envisioned contribution to future generations’. Drawing on the work of Erikson (1950, 1968) and Becker (1973), McAdams (1988: 276) presents generativity or caring for future generations as comprising a two-stage process, through which actors first engender a product or project and then offer it to a community that will reap some benefit from it. In this way, ‘the generated extension of the self [is granted] an autonomy of its own such that the product, which becomes a gift, is both *me* and *not me*’. The identity narratives of entrepreneurial philanthropists, who create products or businesses that they subsequently use to benefit their chosen communities in a targeted fashion, might be expected to articulate and reflect this notion of generativity.

Philanthropists themselves are also subject to influence emanating from the structural contexts and social narratives within which they are located (Downing, 2005; Ezzy, 1998). Individual identity is ‘socially constructed’ (Jenkins, 1996: 20). Turner et al. (2002) observe that organizational leaders are increasingly expected to present themselves in a moral and ethical light. Quasi-hegemonic societal discourses act on wealthy entrepreneurs, urging them to embrace particular moralities by assuming ‘publicly available “personas” or social-identities’ (Watson, 2008: 127). They are thereby encouraged to ‘align with … societally prescribed selves’ by becoming philanthropic (Brown, 2014: 10–11). These discourses concern: the need for *fairness in society*, a key cross-party tenet of the 2010 UK election campaign (Wang et al., 2011); the obligation for the rich *to do their ‘bit’* to help others less fortunate; and the requirement to show solidarity with those who are struggling financially by demonstrating *we are all in this together*. These discourses became more strident in the UK in the period of austerity following the financial crisis. The stories of individual philanthropists are thus embedded in wider canonical discourses with which they resonate, according to which their identity claims are calibrated.

Whereas some commentators regard charitable giving as entirely selfless (Acs and Philips, 2002; Boulding; 1962; Radley and Kennedy, 1995), Bekkers and Wiepking (2011) argue instead that there is a reciprocal element to philanthropic contributions, but that it is indirect and value-oriented rather than overt. This chimes with Phelps (1975: 2), who views altruism as deferred, self-interested investment – a ‘quid for a more implicit and conjectured quo’. As such, the funds deployed by entrepreneurs turned philanthropists, who as entrepreneurs seek to change society, are intended to make a difference, invested in ventures of their choice over which they exercise control (Bandura, 1997).

Ibarra and Barbulescu (2010) argue that stories that follow familiar plots are more persuasive than those that disregard cultural rules. The oral tradition of storytelling often features the journey motif, exemplified by Homer’s epic poem of Odysseus’s voyage. This reveals the journey as representing ‘pain and punishment, the expiation of guilt and the desire to return’ while affording the potential for discovery, exploration and personal change (Gherardi, 2004: 35). Similarly, charitable endeavours may prove to be a route to personal transformation (Tsoukas and Chia, 2002). Authoring philanthropic self-narratives fuelled by the desire to live a better life while improving future conditions for others stimulates new patterns of meaning (Creed et al., 2014). Philanthropic stories ‘emplot’ the life stories of entrepreneurial philanthropists by making the chronological appear causal, generating a coherent narrative that makes sense of their lives (Czarniawska, 1998; Downing, 2005; Fiol, 1989; Martens et al., 2007). Giddens (1991: 54) observes that a person’s identity lies primarily ‘in the capacity to keep a particular narrative going’. This notion of identity as a narrative to be maintained over time while admitting change chimes with the journey metaphor – a sustained self-narrative proving vital to navigating ‘the jungle of human existence’ (Erikson, 1968, cited in Gergen, 1991: 38). Storytelling re-frames entrepreneurial philanthropy by engendering localized meanings that are ‘dynamically enacted in relation to pluralist, shifting landscapes’ (Tams and Marshall, 2011: 109).

Brown (2014) recently called for other metaphors to be considered alongside identity work to elucidate identity formation. Here, we propose in response *the journey* as a metaphor for identity-building and adaptation, life being ‘subjectively structured by a sequence’ (Habermas and Bluck, 2000: 750). In a situation where entrepreneurial and organizational careers are increasingly non-linear and peripatetic, philanthropy helps to secure a stable professional identity, driving and simultaneously justifying the ongoing journey and narrative (Hytti, 2005; Karp, 2006). We aim at the same time to extend theoretical understanding of research into the practice of entrepreneurial philanthropy by laying the foundations for a theory of philanthropic identity narratives (Creed et al., 2014; Downing, 2005; Gregg, 2006; Taylor et al., 2014).

## Research process

Our research is situated within the confines of a larger investigation of individual and business giving, both historically and in the present. For the purposes of this article, life-history interviews were sought with wealthy UK-based entrepreneurial philanthropists who had invested a minimum of £1m in philanthropic projects since 2007. Twenty interviews were conducted by members of the research team as opportunity arose (2008–2012), often after a long period of trust-building and negotiation: nine with super-wealthy individuals whose fortunes exceeded £100m, and the remainder with individuals with a net worth between £10m and £100m (see Table 1). The interviews took place in cities across the UK according to the philanthropist’s primary location, including Aberdeen, Edinburgh, Glasgow, London and Newcastle. All interviewees were UK-based: 12 were Scottish, seven were English and one was Welsh. All were white British, with the exception of one Asian British participant. Their ages ranged from 42 to 71, with a mean age of 57; the gender balance was heavily weighted towards men, with just four women interviewees, all of whom practiced philanthropy with their partners. In order to lend additional perspectives, a further 20 semi-structured interviews were conducted with wealth advisers (four interviews), policy-makers (three), public intellectuals (three) and philanthropic foundation executives (10).

**Table 1.** Participants.

Pseudonym	Source of fortune	Philanthropic interest(s)	Philanthropic vehicle(s)
Ahmed	IT	Islamic community and family welfare services, education	Family foundation
Alistair	Recruitment	Education, relief from poverty	Family foundation, personal donations
Cathy	Property	Support for young people, education and skills	Pooled resource foundation
Desmond	IT	Education and international economic development	Family foundation, company foundation, pooled resource foundation
Gareth	IT	Health research, community support	Personal donations
Gerald	Food and drink	Education, community support, culture, heritage, health research	Family foundation, personal donations
Grant	Finance	Enterprise development at home and overseas	Personal donations
Helen	Insurance	Community support, environment, child development, international economic development	Family foundation
Iain	Business services	Education, support for young people, community support, health research	Family foundation, personal donations
Ingram	Food and drink	Community support, child development, environment	Company foundation, community foundation funds, personal donations
Ivor	Energy services	Enterprise development, education and international economic development, support for young people	Family foundation
Jessica	Finance	Child development, relief from poverty, education, environment	Family foundation
Jimmy	Automotive sales	Community support, child development, enterprise development	Personal donations
Julian	Engineering	Education, support for young people, enterprise development	Pooled resource foundation, personal donations
Kyle	House-building	Support for young people, community support	Company foundation, community foundation funds
Laura	Transport	Education, culture, environment, health research	Family foundation, community foundation funds
Miles	Recruitment	Enterprise development, international economic development, support for young people	Company foundation, personal donations
Phil	Finance	Education, public health	Pooled resource foundation
Timothy	Transport	Education, culture, environment, health research	Family foundation, community foundation funds
Tobias	Retail	Education, support for young people, enterprise development, international economic development	Family foundation

The notion that philanthropists might have stories to tell from their experience was sparked early in our data collection during an interview with a community foundation CEO (community foundations being collective vehicles for charitable giving focused on particular geographical areas: Jung et al., 2013; Pharaoh, 2008). The CEO remarked that sharing stories of philanthropic success had benefited his foundation:

People hear other people’s stories of philanthropy from an invited guest, from somebody we’ve brought in through our contacts and who people can be inspired by their story and think: ‘I could be like that. What could I do?’. (Philanthropy professional)

At interview, we posed a range of questions relating to participants’ family and social background, education, entrepreneurial career, philanthropic career, the motivations underpinning these, their methods, practices and interests, and their peer groups and mentors. We followed a pattern of opening with questions concerning their early life, before probing their entrepreneurial career histories, critical turning-points and the transition to philanthropic engagement, including setbacks and highlights, and concluding with questions regarding what philanthropy had come to mean for them. We did not ask participants to recount stories directly from their life histories, but allowed these to emerge naturally. All interviews, typically lasting between 90 minutes and two hours, were recorded and transcribed, and participants accorded pseudonyms to ensure confidentiality.

A first reading of the transcripts highlighted the philanthropic journey – the process by which individual philanthropists became philanthropic – as central to their stories. As we immersed ourselves more in the data (Berg, 2009), further readings and reflection highlighted their expression of the motivations behind their philanthropy and the rewards accruing to them in consequence. This led to the inductive emergence from the data of a typology of five conceptual categories expressing the rewards they claimed to derive from philanthropy (Glaser and Strauss, 1967). These categories are: *giving back* – the practical recognition that the society that nourished you needs to be nourished for others to flourish in turn; *making a difference* – the aspiration to make a direct, measurable contribution to a particular cause; *absolving the self* – freeing the self from guilt caused by possession of inordinate wealth; *joining the club* – making common cause with others of similar disposition in philanthropic endeavours while joining the philanthropic elite; and lastly, *personal fulfilment* – acquiring a sense of personal achievement stemming from improving the lives of others. Further analysis suggested that these five categories might be collapsed into three second-order categories: firstly, *helping others to help themselves*; secondly, the individual’s own desire to *live a better life* (Tams and Marshall, 2011); and thirdly, *generating a legacy of the self from which others might benefit* (see Table 2). These reflected in turn different facets of *generativity* – being oriented both towards the self in engendering an ongoing journey and narrative (self-directed generativity) and towards society, spurring their capacity for agency in targeted communities (socially directed generativity). In conjunction, self- and socially directed generativity were instrumental in generating a legacy of the self (Creed et al., 2014; McAdams, 1988, 1993).

**Table 2.** Rewards of philanthropic engagement.

Logic of engagement	Type of reward	Exemplary quotations
Helping others to help themselves: *Socially directed generativity*	*Giving back*: Practical recognition that the society that nurtured you needs to be nurtured for others also to flourish.	‘I was brought up in a Muslim household and charity, and giving back to the community and contributing to the community is a very large component of that’. (Ahmed)
	*Making a difference*: Aspiration to make a direct and measurable contribution to a particular cause.	‘I just thought, we can really make a difference here … because that’s what entrepreneurs do … What we have to know is what the outcomes are, and we have to be able to measure it just like in any business situation … I’m really determined that I’m going to make a difference’. (Cathy)
Living a better life: *Self-directed generativity*	*Absolving the self*: Freeing the self from guilt caused by possession of great wealth.	‘I hate the thought of my grandchildren or whoever’s grandchildren saying, “Look at these guys, they all took it all for themselves, made a lot of money, what have they left us?”’. (Ivor)
	*Joining the club*: Making common cause with others of a similar disposition in pursuit of the greater good.	‘The feeling of enormous luck that I have … because we get to meet the most fantastic people, some of whom are really very, very high calibre indeed … just the ability to get to know them, and in some cases make friends with some really extraordinary people, is very, very special’. (Gerald)
Generating a legacy from which others might benefit: *Self- and socially directed generativity*	*Personal fulfilment*: Deep sense of personal achievement stemming from changing the lives of others for the better.	‘It’s given me my life … being exposed to philanthropy; I think it’s given my life much more meaning’. (Gareth) (*self-directed generativity*) ‘I wanted to help the people who didn’t have the advantages that I do to make a better use of their lives’. (Iain) (*socially directed generativity*)

To ensure reliability, coding was carried out by two of the researchers, with differences discussed. Within the overarching life-story narratives, individual extemporaneous stories pertaining to the five conceptual categories were searched for and identified, to which story names were ascribed. We put our conceptual categories to the test by hosting, in March 2013, a symposium on ‘Contemporary philanthropy: Learning from research and practice’, attended by 17 philanthropists, 39 philanthropy professionals and 11 researchers. The extensive discussion that ensued prompted us to reconsider our analytical categories. We had tentatively envisaged our categories as hierarchically graded, proceeding step-by-step from *giving back* as the most fundamental to *personal fulfilment* as the peak of benefits philanthropists claimed to derive. Our audience confirmed that these categories ‘rang true’ for them, based on their first-hand experience. However, attendees provided valuable feedback by suggesting we reconfigure these rewards as a typology rather than a hierarchy. After further consideration, we reconceived our analytical categories as a typology of first- and second-order categories to reflect their expressed reality, according to which we recoded our data.

We have sought in the course of this research to remain explicitly reflexive about our own position. Our research into entrepreneurial philanthropy was originally inspired by our work on elites and elite power (Maclean et al., 2006, 2010, 2014), through which arose a fundamental but compelling question that refused to be suppressed, namely: *what do they do with all the money?* This question became more pressing as the recession deepened. Gaining access to elite actors can be tricky (Pettigrew, 1992). However, our longstanding interest in elites facilitated access to philanthropists, achieved at times through existing contacts, enabling us to secure interviews with some who did not normally grant them. This may also have encouraged participants to be more forthcoming with their life stories, thus influencing our results.

We were naturally conscious of the power asymmetries that obtained between ourselves as researchers and the wealthy individuals we interviewed. We were aware of our role in providing an audience for whom philanthropists could ‘perform’ and from whom they could seek validation for their identity claims (Brown, 2006; Ibarra and Barbulescu, 2010). Legitimacy is arguably located less in philanthropic giving than in ‘a *relationship* with an audience’ (Suchman, 1995: 594). The construction of self-narratives is an ‘integrally political and power-laden process’ (Ezzy, 1998: 250; Goffman, 1959), even if actors seek to disguise this. Viewed in this light, the accounts authored by participants can be understood as self-serving, political acts, through which they sought to safeguard their narrative identities (Brown et al., 2008). We see ours as a story of stories, part of a broader societal narrative that is both an effect of power and the product of specific representational strategies – which must appear disinterested, ‘on the hither side of calculation’ (Bourdieu, 1977: 214), to succeed – through which participants affirmed their dedication to philanthropic engagement. In what follows, we synthesize our findings on the philanthropic journey and examine our five integrative categories, illustrating these with stories told at interview.

## Identity and the philanthropic journey

In expressing their philanthropic career as a *journey*, participants tapped into romantic notions of the quest. As with Odysseus’s voyage, the road may be long and strewn with difficulties. As the traveller proceeds, he or she may encounter surprises and pitfalls. At times forces may enable the protagonist (e.g. the sudden acquisition of affluence) or block the path (e.g. lack of personal experience and understanding), inducing self-doubt and changes of direction (Fiol, 1989). In such circumstances, resilience and perseverance matter, and meeting a guide *en route* to point the way ahead may be invaluable. A breakthrough occurs when the protagonist finds (or conceives of) the object of the quest in the form of tailored projects reflecting personal interests and identities (Fiol, 1989; Zahra et al., 2009). This discovery initiates a new phase of targeted giving and the assumption of donor control to realize social objectives that generate a ‘legacy of the self’, which becomes the ultimate goal (Bandura, 1997; McAdams, 1993: 227). Through these travails, the self may emerge altered from the experience, but with a seemingly more secure sense of identity, perhaps returning to the starting-point of the quest and understanding it better. We now examine the individual stages of this synoptic journey.

### Setting out

The initial stage of the philanthropic journey is characterized by *tentative exploration*. The antecedents to individual journeys were often similar, charitable dispositions often being fostered through prior socialization in the family during childhood (Pratt et al., 2006). As Laura explains:

From my father I got a very strong sense of giving, and I remember when I was quite young, if there was a world crisis, there would be a jam jar on the table at every meal time and we would put pennies into it. We couldn’t afford a lot but it gave me a great sense of giving and feeling that you were making a difference. (Laura)

Other participants exhibited a pre-existing awareness of the developing world, with some travelling to Africa in their youth, and feeling compelled to act upon what they saw. Desmond illustrates this point:

I spent about nearly three years in southern Africa, South Africa. I travelled around Namibia and Botswana … I was always interested in Africa … I came back in 1985, somewhat reluctantly but thinking at 25 I better get a proper job … We saw a lot of stuff in the time that we spent there. (Desmond)

The focus of the early phase of the entrepreneurial philanthropist’s career is nevertheless on business growth and wealth creation, philanthropy being at this juncture an emergent secondary identity complementary to a primary business identity. Economic capital is the starting-point of the philanthropic journey (Bourdieu, 1986; Harvey and Maclean, 2008). Jessica was unusually frank about this: ‘We all want to imagine that the most important part is not necessarily the money, but it is, that is the core of what enables you to … do all those programmes’. Those new to wealth were prone to generosity; as Phil states, ‘they are generally more willing to give it away than people who’ve inherited wealth, because they are confident they can make it again’. Frequently, participants came into wealth abruptly, through selling a business they had built up; this sudden acquisition of affluence being experienced as a ‘landmark event’.

For some, an unexpected consequence of ‘hitting the jackpot’ in this way was having time on their hands. For Gareth, this meant the dissolution of his self-identity as a successful entrepreneur: ‘It was very weird waking up the next day thinking, “What am I going to do?”. So, I basically took some time out.’ Taking time out was not an unadulterated boon:

People used to say to me ‘what do you do?’. ‘I don’t, I’m taking time out …’. I would be at a dinner party and say I was taking a career break or between roles. People would think you were unemployed, so they would just ignore you, and it was a real good lesson. (Gareth)

Here, Gareth expresses the loss of his recognized role as a moment of ‘extreme self-divestiture’ (Ricoeur, 1991: 80). In contemporary society, where the maintenance of identity is crucial (Bendle, 2002), the liminal state he describes of being role-less sparks ideas of time spent ‘wandering in the wilderness’ (Ibarra and Barbulescu, 2010: 143). His identity dislocation recalls Musil’s (1995) notion of the ‘man without qualities’ who, lacking properties, becomes unidentifiable (Beech, 2011; Ricoeur, 1991). This episode confirms pressures stemming from others to play a part in society and hence have a story to tell, Gareth’s loss of words when asked what he did spurring him to do something meaningful with his life.

Other ‘nuclear episodes’ or ‘jolts’ included personal tragedies, like an accident or death of a loved one, which prompted individuals to rethink their life’s course (Dutton et al., 2010: 282). One interviewee, Miles, experienced an ‘epiphany’ on the slopes of K2, when his Sherpa suffered a collapsed lung near the summit. For four days he carried him down the mountain. At one point, he resolved that if he survived, life would change. On day four, dehydrated and frost bitten, he awoke to the face of a young girl who led him to a village, experiencing this deliverance metaphorically as rebirth, after which he forged a new identity centred on an ethic of care as a micro-enterprise lender to small African businesses (Dees and Anderson, 2006; Simola et al., 2010). Miles’s story revolves around a turning point from a previous (presumably unsatisfactory) life to a more fulfilling one. His account, in which he plays the role of hero who overcomes adversity to achieve his mission, is performative, carefully crafted to signal an important moral aspect to his identity (Gabriel, 2000).

### Wayfinding

The second, intermediate stage in the journey entails a period of growth and development, characterized by a continued focus on business interests and wealth management, complemented by a growing determination to improve the lives of others. The overriding *timbre* of this period is *learning and adaptation*, as actors adapt their identities in response to events and encounters through a process of wayfinding (Tams and Marshall, 2011).

Downing (2005: 197) notes that ‘engagement in downfalls, contests, and scams [are] as familiar [to entrepreneurs] as engagement in quest plots’. Participants experienced failure as well as success. Desmond relates one episode whereby anti-retroviral drugs intended for HIV-positive African children were supplanted by placebos and sold for financial gain:

We have had our fair share of disappointments, which range from … kids that we think are running through anti-retroviral treatment for HIV and then you find out that the doctors that you worked with and trusted are actually not giving them drugs. They are giving them placebos … You ask the kids, ‘are you taking your medicine?’, and it is ‘yes, yes, yes’, but they are not getting anything and the drugs that are getting supplied are sold on the black market. (Desmond)

After this setback, the continuation of Desmond’s personal transformation depended on his resilience to recover and move on from his disappointment:

It distorts your ability to move to the next programme, and it takes longer before you do something. So you get very high points and very low points. It still drives me nuts but I don’t dwell on it for quite as long. So part of that is just a maturing process, it is how it is … and it is about kind of moving on with it. (Desmond)

All participants said they learned valuable lessons on their journeys. Many began in reactive mode, responding to demands for financial assistance by random cheque writing. For Julian, it was hard to refuse, ‘because most cases … it’s something you can identify with, and you naturally want to help if you can’. This left him feeling overwhelmed, prompting the insight that it was possible to make things worse; that there was a corresponding need to ‘do it right’; and that this might entail becoming a ‘tough giver’. Not all interviewees abandoned cheque writing on demand, but most came to recognize the need to decide up front which social investments to target. Focusing on select causes allowed them to take control (Bandura, 1997). Discovering the object of their quest in the form of social programmes that matched personal concerns signified a major step forward in the journey (Fiol, 1989; Zahra et al., 2009). As entrepreneurs, their propensity was to apply business principles to social problems, monitoring impacts in selected fields (Dees, 1998). For many, active engagement in social programmes became as important as donating in the first place, shaping their self-identity in a way which, for Miles, had become his ‘life’s purpose’ (Dietlin, 2009).

Given the potential for personal change accompanying philanthropic engagement, it became vital for many to travel the route together with partners and families, engaging in philanthropy as a unit. Jessica puts her finger on this:

We are growing enormously as people in our own understanding of issues and problems and geographies … Then there are definitely changes of how you, particularly because there are two of us, how you do this in a way that we do grow and have the vision together? (Jessica)

Going it alone proved a more arduous experience, given limited understanding of the philanthropic process. As Gareth explains, ‘I felt like I wanted to start giving but I didn’t know how to or who to give to or what way to do it, I hadn’t a clue, *no one teaches you how to be philanthropic*’. We found that a number of participants benefited from a ‘guide’ or mentor on their journey – typically an established philanthropist or wealth adviser who won their trust and indicated how others had successfully navigated the path before them (Gherardi, 2004). Tobias explains the logic behind this:

I went over to New York and knocked on the door of the President of the Carnegie Corporation … He was a great influence in explaining about Carnegie’s principles, and he had also helped Gates, Clinton and various people get their head round the whole idea of philanthropy. So this was a whole new education to me. (Tobias)

Role models point the way ahead while revealing potential personas that actors can experiment with to see if they ‘fit’ their sense of who they are or might become (Ibarra, 1999).

### Home-coming

Entering the final phase, the philanthropic identity becomes more fully developed. This might entail a withdrawal from front-line management to focus on social investments through a disciplined application of business-like philanthropic methods and resources. It is during this mature stage of the journey that participants claim to experience most rewards. Overriding preoccupations at this time include *generativity and succession planning* for the future.

The epic quest story often concludes with a home-coming, whereby the initial lack that propelled the wanderer on a quest is annulled, and the ‘hero’ returns to see the journey’s starting-point in a new light (Gregg, 2006). To quote TS Elliot’s poem *The Waste Land*, ‘the end of all our exploring will be to arrive where we started and know the place for the first time’. The impulse that charity should be concentrated at home was strongly felt, with interviewees regularly investing in their home town, region or *community of origin*, however defined. Iain explains his thinking:

I knew that if I were born [there], I am not sure I could have made my way out with the problems and the lack of aspirations. So my thought on that was, I don’t need to go to Africa … There is too much real poverty and poverty of ambition through too much loss of talent, loss of skills in this country, and that is the best thing to focus on. (Iain)

For most, philanthropy was by now the primary aspect of their identity; without their business identity deserting them entirely. Alistair confirms that becoming philanthropic helped him avoid on retirement the unravelling of identity experienced by Gareth, enabling him to construct a new professional identity equivalent to a second career:

It has given me a second career. I wouldn’t like to be part of this world and just thought of as an employment agent. It is a bit hollow when people ask, ‘What did you do with your life?’. Oh well, I was an employment agent, a great employment agent, but really it gives you another leg to stand on, and if you have lost one you have still got the other. (Alistair)

Looking ahead, the aim shared by many participants was to make their role superfluous so that, as Desmond states, ‘if we walked away or got run over by a bus it would continue’:

One of the things we always try to do … is to understand when we can disappear, because ultimately what we are trying to do is to do ourselves out of a job here. We don’t want to be there forever … seen as one of those guys with money. We are really looking for how do we get out, when do we get out, how would we know when that day has arrived? (Desmond)

Having an exit strategy was therefore, paradoxically, intrinsic to caring for future generations. McAdams (1993: 230) likens this to the notion of the ‘hero’s gift’ (Becker, 1973), which entails caring for a programme sufficiently to let it go, the ‘care and letting go signal[ling] a more communal aspect of generativity’. As Timothy explains: ‘It’s about trying to find ways of curing the problem, but also making sure that the people around the problem begin to change their way of life to stop those sorts of problems happening in the future.’

Generativity involves an element of succession planning, handing over to others to ensure the continuation of charitable programmes in the future. This may entail co-opting offspring onto charity boards. This puts a new complexion on the exercise of control by philanthropists that ultimately they entrust to others, as ‘the generated product is offered as a gift to a larger community … mak[ing] for the continuity, and perhaps even the progressive development, of some aspect of the human enterprise’ (McAdams, 1988: 276). In what follows, we examine more closely the five conceptual categories of philanthropic reward deriving from our data, illustrating these with stories told by participants, and explaining where each fits within the overarching philanthropic narrative.

## Philanthropic rewards

### Giving back

*Giving back* to society, because they are in a position to do so, is something our research subjects deem fundamental (Maclean et al., 2012). Charitable giving is part-and-parcel of the elite equation (Bourdieu, 1977). Giving back makes wealth more acceptable to the public at large, so that, as Kyle observes, ‘it is okay to have done well and then to give something back’. For Desmond, giving back was a sentiment rooted in his upbringing: ‘If you had something you had to share with people … and that was just how things were’.

For many participants, giving back means reinvesting in a community *with which they can identify* because they or their wealth originated there (Boulding, 1962; Johnstone and Lionais, 2004; Jung et al., 2013). Giving back was felt as a motive throughout the journey, but especially in its mature phase, when philanthropists were in a better position to give back by virtue of their accumulated experience, knowledge and wealth. For Timothy, showing solidarity with the north east was a matter of natural justice:

I certainly felt quite strongly … that the wealth that we’d got was created through all the 50ps that myriad Geordies [from Newcastle] and Mackems [from Sunderland] had paid … and basically that’s where the wealth had come from, and that we owed it to that area to put something back. (Timothy)

Gerald echoes this point, stressing the importance of giving back to the communities where the family business garnered its revenues: ‘If Peterborough, as the seat of [large food and drinks company] and its enormous cash flows, has a theme that needs supporting then … we might want to give back to Peterborough because, “thank you very much”.’

Targeting identifiable communities enabled philanthropists ‘to generate *specific* moral capital’ in that area (Godfrey, 2005: 793). As Jimmy opines, ‘you want to operate in a community that would like you to operate in that community’. Ingram tells of pie and peas supper parties he hosted for pensioners in his local area:

We started doing pie and pea suppers for the retired people which were fantastic fun. And the staff enjoyed it, I enjoyed it … and they had a real good party every maybe six months within that community, and that just seemed to me was a nice thing to do, reciprocate and give back something into that community. But, also, it was helping us establish our image and our reputation in the area. (Ingram)

Acs and Phillips (2002: 192) argue that ‘altruism is superior to enlightened self-interest’. Yet, being altruistic does not signify that such actions are necessarily ‘interest-free’ (Suchman, 1995: 579). The *quid pro quo* may revolve around this being the right thing to do (Benkler, 2011; Boulding, 1962), but contributing to the wider social environment impacts positively on reputation, which is socially constructed (Brammer and Millington, 2005; Monroe, 1994). As Godfrey (2005: 777) bluntly puts it, ‘good deeds earn chits’. Hence, the hosting of pensioners’ suppers boosted Ingram’s company’s standing in that community.

### Making a difference

Whereas alms-giving in the past paid little heed to the actual outcomes of redistributed wealth, today’s entrepreneurial philanthropists are concerned with *making a difference*. This motivation strengthened as the journey unfolded and philanthropists became more proactive, particularly when alighting upon the object of their quest where they intuited that a difference could be made. Hence, much of the current discourse on philanthropy revolves around having impact (Dees, 2008; Duncan, 2004; Villadsen, 2007). Being impactful in circumstances that are complex and evolving implies shifting the focus from the intractable to a defined area where a difference may be achieved. To make a difference to a specific social end is still to make a difference. As Kyle articulates, ‘we are never going to be a leader, in the sense that there are some terrific icons to follow, but hopefully we can make a difference’. Cathy sums this up well:

The entrepreneurial me says we should go and do the big thing, but the realist in me says we should go and do something smaller and, actually, have something that works … Sometimes you don’t have to change everything for everything to change. (Cathy)

One example of a small but significant difference to the lives of children in care was related by Julian. This concerned a boy who moved foster homes repeatedly. Each time his possessions were transported in a black bin bag, like garbage. This sparked in Julian the idea of purchasing smart holdalls for local foster children:

There was one boy … he’d had to move more than six times in a year into different schools. When the social worker came to get him … all his clothes got put into a bin bag and his lasting impression was what do you put in a bin bag? You put rubbish. And they wouldn’t let him take his toys or anything with him. And when he went to school, he didn’t have a proper bag or anything, and we’ve got members who, when they heard this, said: ‘Look, we would be happy to provide bags for them all’. (Julian)

Providing bags for children in care may overlook the ‘systemic and structural social inequalities that powerfully shape the lives of these children’ (Swalwell and Apple, 2011: 372), but it does recognize the importance of bolstering their self-esteem so they feel validated. Dees (1998: 56) contends that ‘institutional charity can undermine beneficiaries’ self-esteem and create a sense of helplessness’. For this reason, self-help is high on philanthropists’ agendas, as they seek to dissolve the dichotomy of rich donors and passive recipients to instigate a socially directed generativity aimed at empowering beneficiaries to help themselves (Archer, 2000; Villadsen, 2007). Stories like Julian’s nevertheless throw into relief the acute status differentials between philanthropists and their ‘client base’. The ‘politics of storytelling’ (Ezzy, 1998: 250) are such that the effect of recounting this story is also to boost Julian’s moral capital by revealing him as a compassionate individual who cares about those positioned ‘at the bottom of status systems’ (Snow and Anderson, 1987: 1336).

### Absolving the self

Our third conceptual category is that of *absolving the self*. Radley and Kennedy (1995) argue that ‘gift-giving in society provides its members with a way of “atoning for sins” and easing their conscience’. Experiencing guilt may nevertheless curb self-interest (Wang et al., 2011). The impetus to free the self from guilt increased with wealth accumulation, and was something interviewees expressed more keenly in the mid-stage of their journey, when the contrast between their own and others’ circumstances was especially acute. Grant spoke candidly of his discomfort over rising inequalities:

I find it quite hard to look at the inequality … and not feel that I am making some contribution to it … and there is an element of salving your own guilt or conscience within that, which is not necessarily a bad thing to do, better than staying guilty. (Grant)

Nevertheless, being philanthropic may not be enough to convince others that philanthropists are good people. Higher social status is reputedly linked to unethical behaviour (Piff et al., 2012). Philanthropic engagement generates moral capital only if it is made known (Godfrey, 2005). Philanthropists must therefore *narrate* their philanthropic engagement, employing ‘strategies of persuasion’ that are ‘never ethically neutral’ but tinged with the language of redemption (Creed et al., 2014; Ricoeur, 1988: 249). Several participants draw parallels between themselves and Andrew Carnegie, the archetypal entrepreneurial philanthropist who distributed most of his wealth during his lifetime (Harvey et al., 2011). Phil sees this as key to his identity: ‘I identify strongly with Carnegie; you know, “to die rich is to die disgraced”’. Phil was also motivated by faith, considering it his duty to support ‘the people who Jesus visibly helped all through his life on Earth; the poor and those who couldn’t help themselves, the sick, the homeless’. The weaving of such statements into identity narratives makes claims to ethical justice, countering the dominant economic self-interest maximization perspective found in super-wealthy individuals (Ricoeur, 1988). Phil draws parallels with Carnegie and Jesus, such ‘storied hegemonic impositions’ concealing a tacit objective of securing the approval of the dominated, domination being achieved through consent as well as coercion (Brown et al., 2005: 314; Clegg, 1989).

Such identity claims were not always wholly persuasive (Martens et al., 2007). Tobias recounts how he banked ‘a very large cheque’ just as hundreds of his former employees lost their jobs when he sold his business:

It actually felt terrible, because headquarters were on this site here and we employed an awful lot of people, and I knew those jobs were going to disappear because the business was going to be moved … I knew it was the right thing to do, because the market was getting tougher … But it was still a difficult time in people losing their jobs and stuff. Very difficult! (Tobias)

Tobias’s summation of this episode as ‘very difficult’ leaves unstated any guilt he may have experienced over the redundancies, glossing over any self-interested motivation of financial gain. This story puts in parenthesis the undesirable self who instigated the job losses, blamed on market conditions (Ybema, 2010). The event serves as a turning point, his subsequent turn to philanthropy enabling him to reframe the closing of the business as ‘a redemption narrative of self-renewal’ through which he makes amends for job losses and reaffirms his self-concept as a pillar of the community (Ibarra and Barbulescu, 2010: 146). Confirmation of this identity is solicited in the ‘“looking-glass” of others’ reactions’ to his story (Cooley, 1902; Down and Reveley, 2009: 385). In face-to-face interaction at interview, withholding such assurance is problematic given the significant power (and wealth) differentials at play between actors.

Cathy, likewise, tells of laying off workers, and the need to take responsibility for delivering the bad news herself:

When the chips are down, you have to lead from the front in your organization … If I’ve got redundancies to make in this business, it’s me that has to do it. We just had to make some people redundant … and I just got in my car and drove to tell them.

For Cathy, integrity appears to lie in having the courage to tell employees personally (Gabriel, 2000). Unlike Tobias, she exhibits no awareness of the identity mismatch between charitable giving and axing jobs, the self that issued the redundancies being easily bracketed off so there is no detraction from her ‘preferred’ identity, the two being seemingly unrelated (Down and Reveley, 2009; Pratt et al., 2006; Sacharin et al., 2009).

### Joining the club

Philanthropic engagement sustains relationships in the wider community, being ‘part of a matrix of social relationships that it helps to maintain’ (Radley and Kennedy, 1995: 692). Inseparable from the benefits accruing to entrepreneurial philanthropists through charitable giving is the elite access it affords. Philanthropy in this sense is ‘hugely undemocratic’ (Maclean et al., 2013: 757) – something our interviewees were conscious of to varying degrees. The gulf between the philanthropic elite and its client base is accentuated by new relationships of donor exclusivity instigated through philanthropy (Ostrander, 2007). In return for generosity, donors ‘gain passage through inclusion boundaries’ to join an exclusive club (Ibarra and Barbulescu, 2010: 136; Ostrower, 2002). Achieving membership of the philanthropic elite marks a key role and identity transition. Once ensconced as members, philanthropists gain validation for their narrative accounts from other powerful actors, confirming their new identity.

Joining the club was a reward experienced by philanthropists in the more mature stage of the philanthropic journey, when time is more plentiful and they can afford the ‘entrance fee’ and subscription required for membership, that Cathy describes as having to ‘pay to play’. Conforming to the normative discourses that encourage wealthy entrepreneurs to become philanthropists is thus beneficial in itself, boosting social capital. Jessica confirms that philanthropy has given her ‘a seat at the table’ alongside high-status individuals. Cathy enthuses about the contacts she has made through invitations to prestigious social events, including the Clinton Foundation. At one conference attended by ‘70 heads of State’, one speaker took her under her wing:

She’s been Woman of the Year; she went to India with Mother Theresa. Anyway, she adopted me, completely. I took her to the cocktail party and … all these people coming up to her, of course, ‘This is my friend Cathy’ … I was meeting all these, like, unbelievable people. It was like one of the most amazing nights of my life! (Cathy)

Self-narratives extend the benefits of prized connections. Name-dropping is a self-conscious political act designed to produce power effects (Ezzy, 1998). On the flip side, there remains a ‘threat of expulsion’ from the group should philanthropists cease to act on the discourses that exhort them to do their bit; implying ongoing ‘elements of coercion’ to conform even at this elite level (Boulding, 1962: 62, 63).

Tobias has also gained from rubbing shoulders with the philanthropic elite, which has opened doors and generated business opportunities:

There are people that I meet through philanthropy who we’ve done business with that we would never have met if it was a purely business relationship, and there is a huge interest in philanthropy and therefore it opens a lot of business doors for us as well. (Tobias)

This extract suggests that Tobias has benefited financially from his philanthropy, exemplifying the ‘competitive advantage of social orientation’ identified by Dees and Anderson (2006: 56). This underlines the self-interest that infuses philanthropic engagement and the social usefulness of commercial endeavours, questioning ‘the simple model of rational self-interested behaviour … central to economic theory’ (Dees and Anderson, 2006: 59).

### Personal fulfilment

Our final conceptual category concerns personal fulfilment. Tsoukas and Chia (2002) suggest that by striving to experience reality directly, individuals can create new configurations of meaning (Benkler, 2011; King, 2004). Archer (2000: 319) concurs, asserting: ‘It is … through dedicating ourselves to the subjects and objects of our caring that we make our mark upon reality’. The impulse to do so may be all the more pressing for those who materially ‘have it all’ (Giacalone et al., 2008). As Julian puts it:

You can only have so many cars, so many aeroplanes, so many boats, houses; you don’t need the money … The other angle is that if you can … help locally … then I think it gives you a feeling of satisfaction and the motivation to do better. (Julien)

The reward of personal fulfilment is more likely to be experienced in the final stage of the journey, when individuals have most latitude in determining how they spend their time and money. In this regard, Ingram explained how as a boy he enjoyed fishing, sparking an interest in science he was unable to pursue. In semi-retirement, he became involved in River Trusts, to whose service he devotes three days’ volunteer time per week, experiencing at first hand pleasures forgone as a boy:

I was really interested in nature as a child, spent all my childhood running round with a jam jar and fishing net. I had always been … frustrated that I’d never had any sort of scientific education at all. And my initial interest came through fishing and being in the countryside and by rivers where you will see a lot of wildlife … and then I got involved with the River Tweed Commissioners which was absolutely fascinating. (Ingram)

Ingram’s involvement in river management enabled him to experience activities that otherwise would have remained a closed book, spurring him to do more for the environment.

Self-fulfilment entails obligations towards the collectivity, transcending the personal or private sphere to embrace ‘a form of social duty’ (Villadsen, 2007: 320). This relates in part to the opportunity to author a new script for others (Ricoeur, 1995); as Phil puts it, ‘the thing that is most important to me is to help each person to fulfil their potential in life’. To this end, Desmond explains his wish to replace the ubiquitous picture of the starving African child, now taboo, with the positive image of a young, educated woman (Ybema, 2010). He has a particular individual in mind:

There is a girl, Lucy, who was at an orphanage in Nairobi. Lucy is over here at university studying Business … She has always said that what she wants to be is the President of Kenya. She has been there, an opportunity created, and her eyes opened to a whole bunch of things. She is going back and she is going to be President of Kenya, and I tell you something, you wouldn’t want to bet against her. (Desmond)

Wang et al. (2011: 644) write that ‘the push to maximize gains does not include a stopping rule’. However, as the above extract illustrates, parallel to achieving personal goals is the aspiration to help actualize possibilities for others, epitomizing a form of socially directed generativity centred on caring for others in the future, whose expression comes to the fore in this story (Cruikshank, 1999).

## Discussion and conclusion

Entrepreneurial philanthropists are embedded in wider canonical discourses that influence how they narrate their identities to express solidarity with deprived communities and those ‘at the lowest reaches’ of society (Johnstone and Lionais, 2004; Snow and Anderson, 1987: 1336). Hence, philanthropic self-narratives shed light on the fashioning of positive identities in response to broader societal discourses that act on entrepreneurs to become philanthropic (Gregg, 2006; Kornberger and Brown, 2007; Martens et al., 2007). Philanthropic engagement enables entrepreneurs to progress towards a desirable self by performing something worth remembering, through which they seek to absolve themselves from guilt caused by wealth accumulation (Dutton et al., 2010; McAdams, 1988). Their identity narratives are therefore imbued with generativity and nuanced with undertones of redemption-seeking (Creed et al., 2014; McAdams, 1993).

Entrepreneurs’ personal trajectories are expressed by them (and constructed by us) as *journeys* to a new reconstructed self-identity, through which they create new meaning (Brown, 2014; Gregg, 2006; Grey, 1994; Watson, 2008). A breakthrough occurs when actors alight upon (or conceive) the object of their quest in the form of projects targeting specific social objectives that give focus to their emergent self-identities (Dees, 1998; Fiol, 1989; Zahra et al., 2009). Finding what they have been searching for does not signify the end of the quest, but rather the start of a new phase typified by targeted giving and the assumption of donor control (Bandura, 1997). Social agency entails ‘unfolding, ongoing processes’ whereby actors encounter unfamiliar obstacles *en route* and learn to traverse these (Emirbayer, 1997: 289). The exercise of agency combines with the unfolding journey – a marriage of willpower and ‘waypower’ (Luthans et al., 2004: 47) – to produce change. The process of becoming philanthropic thus effects a ‘reconstitution of the political at the level of the self’, fostering a new socially generative self-identity (Cruikshank, 1999: 88).

The low level of charitable giving in the UK, where those earning above £200K annually give just £2 for each £1000 they earn (*Philanthropy Review*, 2011), highlights the need for philanthropists to broadcast their stories to exhort other rich actors to follow suit. These stories are skilfully narrated, but they are also carefully targeted. This is helpful because social change tends to be instigated in the semi-organized field (Ahrne, 1990). Given the extent of unmet social needs, focusing on bespoke projects tailored to specific objectives makes sense; as Cathy suggests, ‘do something smaller and, actually, have something that works’. This recalls Candide’s insight from Voltaire’s (1759/1997) satire of the same name, which concludes with the ‘hero’ returning from his quest to declare ‘we must cultivate our garden’ as a practical response to intractable issues.

We make several contributions to extant literatures. First, in the pre-paradigmatic field where entrepreneurship intersects with philanthropy (Nicholls, 2006; Taylor et al., 2014), our article develops theoretical and empirical understanding of the involvement of wealthy entrepreneurs in socially transformative projects by offering a foundational theory of philanthropic identity narratives (Dees, 1998; Dees and Anderson, 2006; Taylor et al., 2014). Most importantly, we show that these identity narratives are structured according to the metaphorical framework of *the journey*, through which actors envision and make sense of personal transformation over time (Lakoff and Johnson, 1980). As Habermas and Bluck (2000: 751) suggest, ‘One way to explicitly thematically integrate a life narrative is by reference to a central metaphor’. We respond to Brown’s (2014) call for fresh identity tropes to supplement that of identity work by proposing the journey as a valuable metaphor for conceptualizing and ordering narrative identities in entrepreneurial and organizational careers as individuals navigate different kinds of terrain (Ahrne, 1990). The social landscape is labyrinthine and ‘Everybody is an explorer of his own everyday world’ (Ahrne, 1990: 75). Individual careers are increasingly itinerant, and the stages on which they play out are not static but evolving. The journey provides a helpful diachronic narrative template that knits together individual role and career transitions to bridge past and present, whose changing contours and contexts are given explanatory coherence by an overarching life story (Habermas and Bluck, 2000).

Second, we add to the expanding literature on narrative identities by making a contribution to literature concerned with harnessing agentic self-narratives for societal change (Creed et al., 2014; Downing, 2005; Gregg, 2006; McAdams, 1988, 1993). By accentuating individual agency and sense of life purpose, such narratives empower actors to generate a legacy of the self that we demonstrate to be both self- and socially oriented. Ontological questions concerning how to live a good life loom large for those who ostensibly ‘have it all’ (Giacalone et al., 2008). What matters most is *keeping the narrative and journey going* by authoring new self- and socially generative chapters (Giddens, 1991). Ultimately, it is only by making the self available to others in what McAdams (1993: 332) calls a ‘generative synthesis of agency and communion’ that a life-validating legacy of the self can be fashioned (Ahrne, 1990; McAdams, 1988: Ricoeur, 1995). We show this legacy to be both narrative and material in nature, forged through identity storytelling and future-directed socially generative projects.

The journey motif casts light on the protracted, arduous nature of entrepreneurial philanthropic engagement as an ongoing accomplishment, but also hints that it may culminate in and be crowned by rewards. We make an empirical contribution by proposing a typology of the rewards philanthropists claim to derive from their giving, as gleaned from their individual accounts. We discern these as fivefold: giving back, making a difference, absolving the self, joining the club and personal fulfilment. These stem, in turn, from twin logics of helping others to help themselves, and the individual’s desire to lead – and *be seen to lead* – a more meaningful life by shaping a better future identity (Bruner, 1990). Both logics are informed by generativity, propelled by the desire to produce ‘meaningful and long-lasting life products’ (McAdams, 1988: 277) as part of an ongoing narrative and journey.

Finally, our study has implications for actual and prospective philanthropists, through which we make a contribution to practice. We suggest there is an ordered logic to the philanthropic journey that might inform the practice of emergent philanthropists and philanthropy professionals (Gherardi, 2004). Giving back and making a difference to communities of origin are powerful motivators for entrepreneurial philanthropists (Peredo and Chrisman, 2006). The role of a ‘guide’ may ease the transition from entrepreneur to philanthropist; in this regard, training for would-be guides may be beneficial (Ragins et al., 2000). As with business ventures, entrepreneurial philanthropists should invest only in what they can grasp at first hand; only by understanding their chosen area of intervention can they hope to have impact (King, 2004). Inspirational self-narratives help philanthropists to boost resources for philanthropy, marking a trail for others to follow; hence, philanthropists themselves make the best *advocates* for philanthropy (Lounsbury and Glynn, 2001; Martens et al., 2007). Having access to wider philanthropic circles, and sharing experiences, is helpful in refining practices and increasing engagement (Eikenberry, 2006; Ostrower, 2002). Finally, becoming an active philanthropist puts the individual (or couple or family unit) in the driving seat in terms of exercising control over social investments (Bandura, 1997; Ostrander, 2007).

The limitations of the present study include the fact that only British-based entrepreneurial philanthropists participated in this research. To address this, we intend our future work to assume an international focus. To illuminate entrepreneurial philanthropy further, a comparison set of entrepreneurs who were not philanthropists would have been welcome. This might examine why wealth propels some entrepreneurs into philanthropy but not others; it is entirely possible that wealthy entrepreneurs who do not practice philanthropy believe they are helping others by providing work as ‘job-creators’. Accessing such a sample may be problematic, because the wealthy prefer to be seen in a favourable light, and to admit they did not give would leave a negative impression (Brown and Jones, 2000; Schervish et al., 2005). An alternative comparison set might be provided by social entrepreneurs engaging in the non-profit pursuit of social objectives, who might be expected to exhibit strong charitable dispositions but a reduced interest in personal wealth creation (Dees, 2008). Such comparison might elucidate what separates social entrepreneurs from entrepreneurial philanthropists, other than their financial resources, and how their life narratives diverge (Dees and Anderson, 2006). This forms an agenda for future research.

We have demonstrated here that storytelling and action are closely aligned (De Certeau, 1984; Downing, 2005; Ricoeur, 1981). The journey metaphor we employ emphasizes the ‘“waypower” dimension of hope’ (Luthans et al., 2004: 47), which enables actors to feel that they are making progress in their life stories (Carlsen and Pitsis, 2009; Dutton et al., 2010). The re-storying of philanthropy underlines the role agentic self-narratives can play in social change more broadly (Downing, 2005). The stories philanthropists tell are not just bound up with the project of the self or with self-legitimation (Grey, 1994; Lounsbury and Glynn, 2001); they are also concerned with regenerating communities by targeting specific social objectives (Dees, 1998; Dees and Anderson, 2006), through which actors generate a legacy of the self (Creed et al., 2014; McAdams, 1988, 1993). In shifting the focus from the universal to the feasible, philanthropic identity narratives demonstrate the potential of human agency to ‘react back powerfully and particularistically’ (Archer, 2000: 318) to create new loci of responsibility in a profoundly unequal society.
